# Trauma-related mortality in a European region with an intermediately mature trauma system: a comprehensive population-based analysis

**DOI:** 10.1007/s00068-025-03043-x

**Published:** 2026-01-13

**Authors:** Amélie Marie Le Gall Travert Dit Neret, Gonzalo Tamayo Medel, Iñaki Bilbao Villasante, Idoia Sainz-Trápaga Sáez de Villarreal, Jon Arrieta Pérez, Alberto Martínez Ruiz, Gorka Prieto Agujeta

**Affiliations:** 1 Emergency Medical Services of the Basque Health Service – Osakidetza, Bilbao, Spain; 2 Department of Surgery, Radiology and Physical Medicine, University of the Basque Country UPV/EHU, Bilbao, Spain; 3 BioBizkaia Health Research Institute (IIS BioBizkaia), Baracaldo, Spain; 4 Department of Health, Basque Government, Vitoria, Spain; 5 Department of Anesthesiology, Intensive Care and Pain Therapy, Cruces University Hospital, Baracaldo, Spain; 6 Basque Institute of Legal Medicine, Bilbao, Spain; 7 Department of Communications Engineering, University of the Basque Country UPV/EHU, Bilbao, Spain

**Keywords:** Wound and injuries, Mortality, Cause of death, Autopsy, Emergency medical services.

## Abstract

**Background:**

Understanding the epidemiology of trauma-related mortality is essential to guide quality improvement and optimize trauma system performance. However, the absence of comprehensive regional registries often limits accurate assessment. This study aimed to characterize trauma-related deaths in Biscay (Spain), a European region with an intermediately mature trauma care system, including both prehospital and in-hospital deaths.

**Methods:**

A retrospective, population-based observational study included all trauma-related deaths in 2019 and 2022. Data were obtained from forensic autopsy reports and cross-referenced with clinical registries from the Basque Health Service. Variables analyzed were demographics, injury mechanisms, severity scores (AIS, ISS, NISS), ASA-PS classification, medico-legal intent, and physiopathological cause of death. Years of potential life lost before age 70 (YPLL70) were calculated.

**Results:**

A total of 313 trauma-related deaths were recorded: 151 in 2019 and 162 in 2022. Median age was 72 years (P25–P75: 52–84), and 67% were men. Low-energy falls accounted for 41% of cases and high-energy falls for 30%. The medico-legal intent was mainly unintentional (69%), followed by suicide (29%). Traumatic brain injury (47%) and massive hemorrhage (29%) were the leading physiopathological causes. Two distinct profiles emerged: young adults sustaining high-energy trauma with severe injuries, and older adults dying after low-energy mechanisms.

**Conclusion:**

This study highlights a dual epidemiological pattern of trauma-related mortality in a region with an intermediately mature trauma system. Adapting trauma care pathways to address both high- and low-energy trauma, particularly in an aging population, may improve efficiency, equity, and clinical outcomes.

**Supplementary Information:**

The online version contains supplementary material available at 10.1007/s00068-025-03043-x.

## Introduction

Trauma remains a major public health problem and is the leading cause of mortality, morbidity, and long-term disability in adults under 45 years of age. According to the 2021 Global Burden of Disease (GBD) report [1], traumatic injuries caused 4,343,697 deaths worldwide (6.4% of all deaths) and accounted for 9.67% of years of life lost (YLL) due to disease.

Accurately quantifying the burden of trauma is difficult because the concept of “trauma” does not appear explicitly in the International Classification of Diseases (ICD). Instead, it is embedded within the broader category of “external causes of mortality” (ICD-10-ES, 2nd edition, 2018: S00–T98), together with asphyxiation, drowning, poisoning, burns, frostbite, and complications of medical and surgical care [2]. Furthermore, this classification system groups deaths by medico-legal intent—such as suicide, homicide, or unintentional injury—without distinguishing the underlying mechanisms of injury.

According to the Spanish National Statistics Institute (INE), between 7,262 and 11,294 trauma-related deaths were estimated in Spain in 2019 after excluding non-traumatic causes from external mortality categories [3]. This corresponds to 1.7–2.7% of all deaths among individuals aged over 14 years, depending on whether suicides, homicides, and events of undetermined intent are included. Among individuals aged 15–39 years, between 757 and 1,604 deaths were reported, suggesting that trauma accounted for 16.1–34.2% of all deaths in this age group.

While this epidemiological classification serves important public health purposes, it has limited pathophysiological relevance and is insufficient for evaluating or improving healthcare processes. In contrast, trauma registries—which provide structured data on injury mechanisms, clinical management, and outcomes—are essential tools for quality assurance and patient safety [4]. Understanding the epidemiology and caseload of trauma is a necessary first step in designing effective continuous quality improvement strategies within trauma systems.

The establishment and progressive maturation of trauma systems have consistently been shown to improve outcomes for severely injured patients [5–10]. However, most studies focus on in-hospital data, while prehospital deaths—often representing a substantial proportion of trauma-related mortality—are rarely analyzed [11, 12]. Their exclusion limits our understanding of the full spectrum of trauma burden and hampers system-level improvement.

In this context, we conducted a comprehensive population-based study including all trauma-related deaths, both prehospital and in-hospital, in Biscay (Spain). Our objective was to provide an in-depth characterization of trauma-related mortality from a healthcare system perspective, to identify predominant injury mechanisms and physiopathological patterns, and to inform future strategies for improving trauma care organization and outcomes.

We hypothesized that analyzing trauma-related deaths through the integration of autopsy and clinical databases would allow a better understanding of the true burden of trauma-related mortality and the identification of distinct physiopathological patient profiles.

## Methods

### Study design and setting

We conducted a retrospective, population-based observational study including all trauma-related deaths that occurred in Biscay (Spain) during two one-year periods: January to December 2019 and January to December 2022. The years 2020 and 2021 were excluded to avoid statistical distortion caused by the COVID-19 pandemic [13]. Biscay is a province with a population aged over 14 years of 992,931 in 2019 and 997,204 in 2022, with 52% females.

According to the World Health Organization (WHO) Trauma System Maturity Index [14]. Biscay is classified as a region with an intermediately mature trauma system (Table 1). Prehospital care is coordinated through a single dispatch center accessible via the European emergency number 112, managing a medical transport network composed of 67 basic life support ambulances, 7 nurse-staffed advanced life support ambulances, 5 physician-staffed advanced life support ambulances, and 1 Helicopter Emergency Medical Service (HEMS). The covered area spans approximately 2,217 km² and includes both urban and rural zones. The hospital network comprises five centers of different levels (I to IV) and a single forensic pathology service. Patients aged 15 years or older are treated as adults.

**Table 1:** World health organization trauma system maturity Index Adapted from the WHO [14]. Levels corresponding to the current situation in Biscay are highlighted in bold italics EMS: Emergency Medical Service; EsTC: Essential Trauma Care

	Level I	Level II	Level III	Level IV
Prehospital Trauma Care	No mapping of prehospital resources No formal EMS, unavailability or duplication of prehospital services No defined communication system	Prehospital resources are identifiable No coordination between public and private providers of prehospital care No universal access number, weak links of communication	Formal EMS present Universal Access Number available Coordination seen between various agencies for prehospital care delivery Well defined communication	***Formal EMS controlled by a lead agency*** ***National universal access number*** ***Legislative mechanism in place to govern EMS and allow universal coverage***
Education and Training	No identified health personnel to offer primary trauma care in community	Identified health personnel in the community for emergency trauma care No definite training requirement for health workers or ambulance personnel	***Health professionals and paramedics are trained in provision of emergency trauma care*** ***Training courses are available for trauma education***	Educational standards and training for emergency trauma care providers laid down Licensing and renewal norms for different levels of paramedics are in place
Facility-based Trauma Care	Role of secondary and tertiary facilities unclear Health facilities lack human and physical resources No clear referral linkages	Roles of various health care facilities are clear Referral linkages are present No documentation or needs assessment of facilities in line with EsTC guidelines No lead agency in the system	***Health facilities in the systems are assessed in line with EsTC guidelines*** ***Guidelines and documented human and physical resources are available round the clock*** ***Lead agency present***	Mechanism of hospital verification and accreditation is in place through Ministry of Health or professional bodies Lead agency established with mandate to supervise trauma care
Quality Assurance	No injury surveillance or registry mechanism in place to get comprehensive data	Injury data available but no formal attempts to document and analyze the data No initiative for Quality Assurance program	Basic Quality Assurance programs in line with EsTC guidelines Guidelines are in place	Formal Quality Assurance programs are in place and are mandated in prehospital and facility-based services

### WHO trauma system maturity index

#### Case identification and inclusion criteria

All deaths classified by forensic experts as violent (non-natural) were included when trauma was considered the primary cause of death, even if death occurred up to six months after the initial event due to complications or treatment limitation decisions. Cases of isolated burns, suffocation, or drowning were excluded.

For each case, forensic data were cross-referenced with clinical information obtained from the Basque Health Service’s electronic medical records (Osabide and Movipen), as well as the IntelliSpace Critical Care and Anesthesia (ICCA) and Euskarri information systems. This comprehensive data linkage enabled the identification of both prehospital and in-hospital trauma-related deaths.

#### Ethical considerations

The study was approved by the Research Ethics Committee on Medicinal Products of Euskadi (CEIm-E), in accordance with Spanish Law 14/2007 on Biomedical Research, the ethical principles of the Declaration of Helsinki, and other applicable regulations.

#### Variables and definitions

The following variables were analyzed: age, sex, score in the American Society of Anesthesiologist Physical Status (ASA-SP) before trauma, medico-legal intent (unintentional, suicide, or homicide), type of injury (penetrating or blunt), mechanism of injury according to the “Utstein Style for Trauma” [15], severity scores (Abbreviated Injury Score [AIS], Injury Severity Score [ISS], and New Injury Severity Score [NISS]), the most severely affected anatomical regions according to AIS, the number of severely affected regions (AIS > 2), and the cause of death.

Injury severity was assessed using a simplified and validated version of the AIS [16]. ISS was calculated as the sum of squares of the three highest AIS scores in different body regions, ranging from 1 to 75. According to WHO and American College of Surgeons Committee on Trauma (ACSCOT) criteria [4], deaths were classified as Preventable (PD) when probability of survival (Ps) > 50% or ISS < 20, Potentially preventable (PPD) when Ps = 25–50% or ISS = 20–50, and Non-preventable (NPD) when Ps < 25% or ISS > 50.

The ASA-PS scale was used as a proxy for pre-injury comorbidity burden, given its validated predictive value for post-trauma mortality [17]. Age groups were defined according to the pattern observed between age and ASA-PS distribution.

The physiopathological cause of death was selected from predefined categories at the discretion of clinical or forensic investigators based on the most severe injury at the time of trauma. Cases involving multiple major injuries were classified as “multiple trauma.”

Years of potential life lost (YPLL^70^) were calculated using the Romeder and McWhinnie method, subtracting age at death from 70 years. Crude and age-standardized mortality rates were computed using the 2013 European Standard Population as reference [18].

### Statistical analysis

Statistical analysis were performed using *R* version 4.5.1 and Microsoft Excel. Continuous variables were described as medians and interquartile ranges (P25-P75), whereas categorical variables were expressed as frequencies and percentages. Group comparisons were conducted using the non-parametric Kruskal–Wallis test for continuous variables and the Chi-square test for categorical variables, with 2,000 simulations to correct for small sample bias. A p-value < 0.05 was considered statistically significant. Results were rounded to the nearest integer for clarity.

The primary outcome was the quantification of trauma-related deaths and years of potential life lost. The secondary outcome was the description of epidemiological and clinical characteristics of trauma deaths stratified by age group.

## Results

**Fig. 1:**
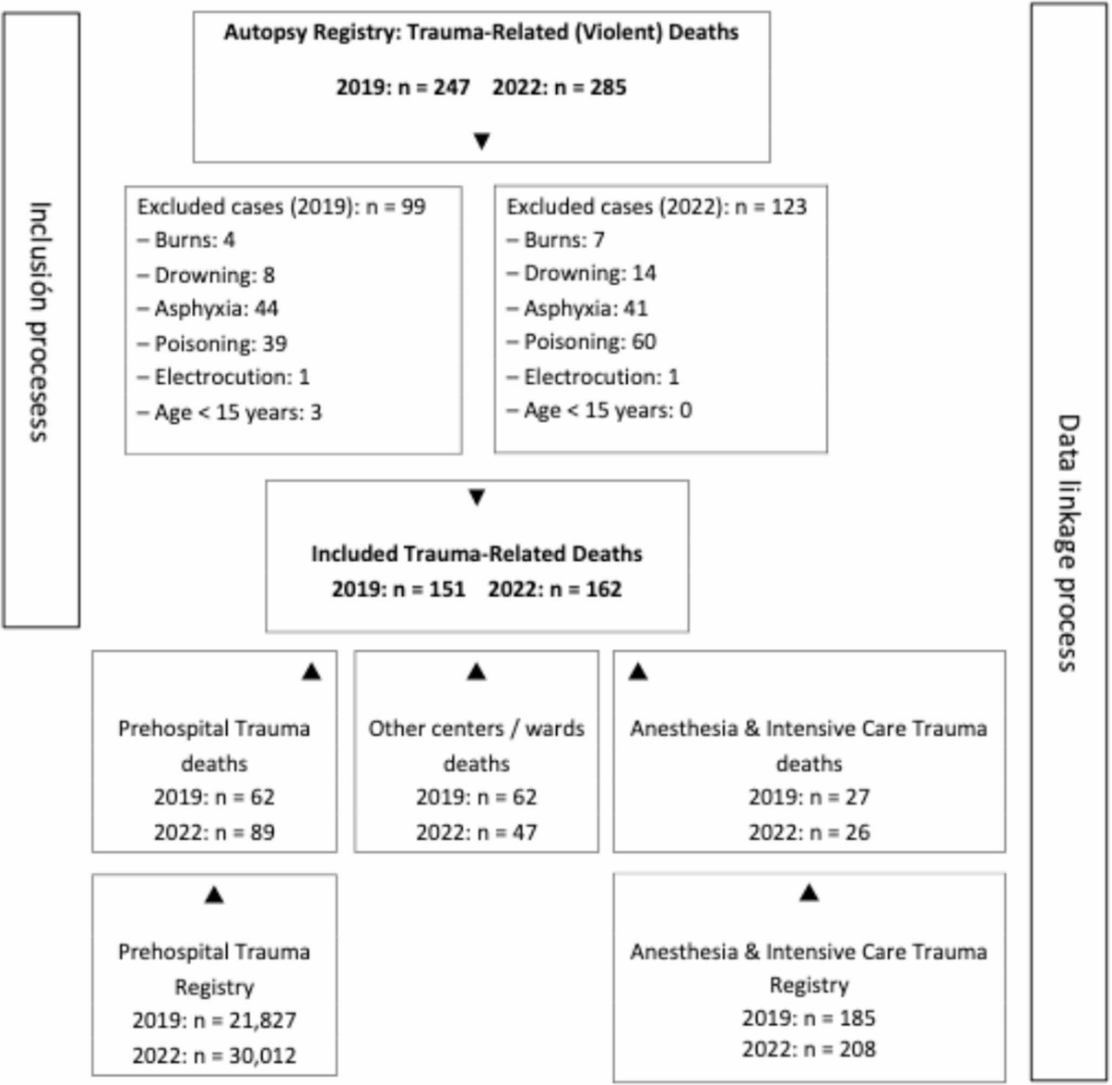
Flowchart illustrating data sources and the inclusion/exclusion process for trauma-related deaths in Biscay (2019 and 2022)

A total of 313 trauma-related deaths were included: 151 in 2019 and 162 in 2022 (Fig. 1).

The crude mortality rate (CMR) associated with trauma was 15.2 and 16.3 per 100,000 inhabitants aged over 14 years in 2019 and 2022, respectively, according to Eustat census data [19].

Women accounted for 30% (*n* = 46) of deaths in 2019 and 35% (*n* = 57) in 2022, corresponding to CMRs of 8.9 and 10.9 per 100,000 women, and age-standardized rates (2013 European standard population) of 7.4 and 9.1 per 100,000, respectively. Men represented 70% (*n* = 105) and 65% (*n* = 105) of deaths in 2019 and 2022, with CMRs of 22.2 and 21.8 per 100,000, and age-standardized rates of 18.6 and 18.3 per 100,000, respectively. The overall median age was 72 years (P25-P75: 52–84) (Supplementary Table 1).

### Age distribution

**Fig. 2:**
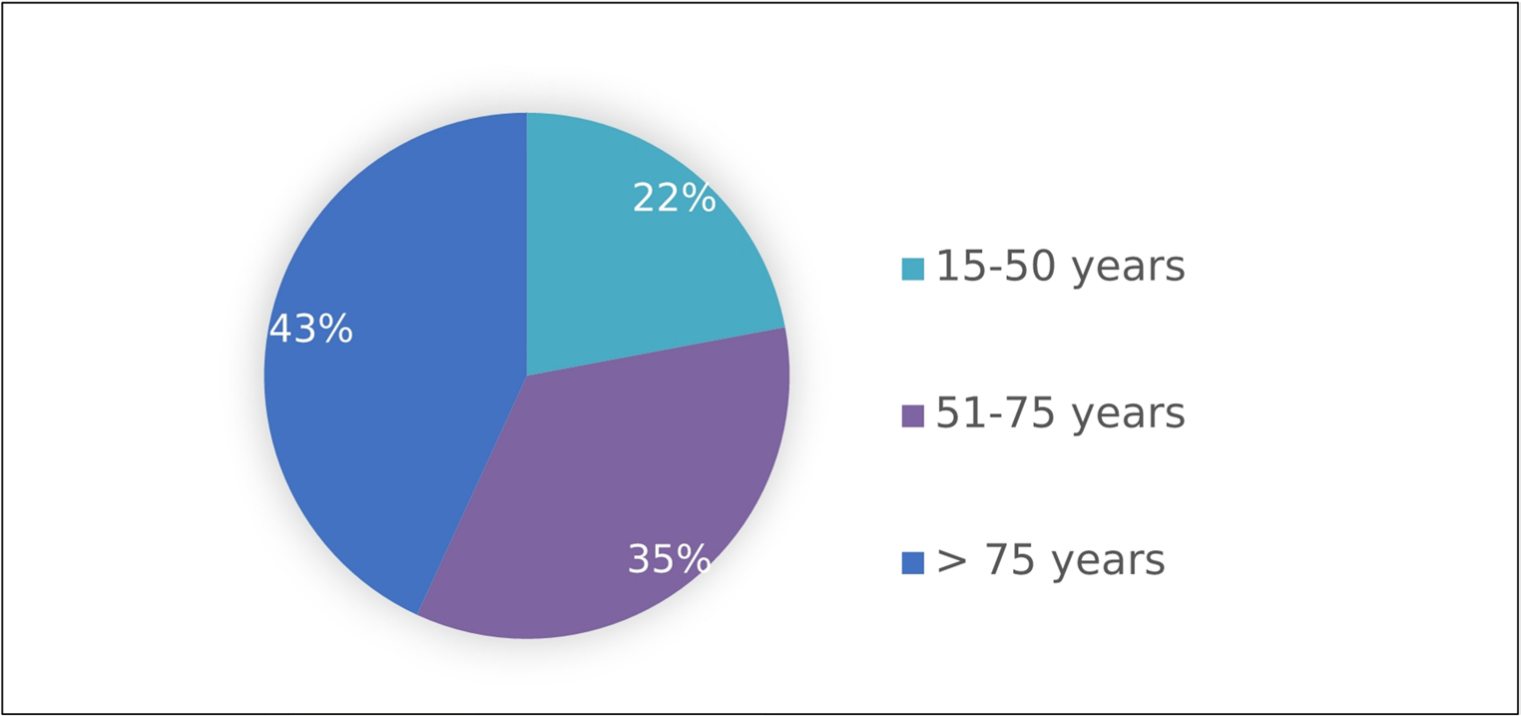
Age distribution in trauma-related deaths

 Deaths occurred in 22% (n = 69) of cases among individuals aged 15–50 years, 35% (n = 109) in those aged 51–75 years, and 43% (n = 135) in those over 75 (Figure 2).

**Fig. 3:**
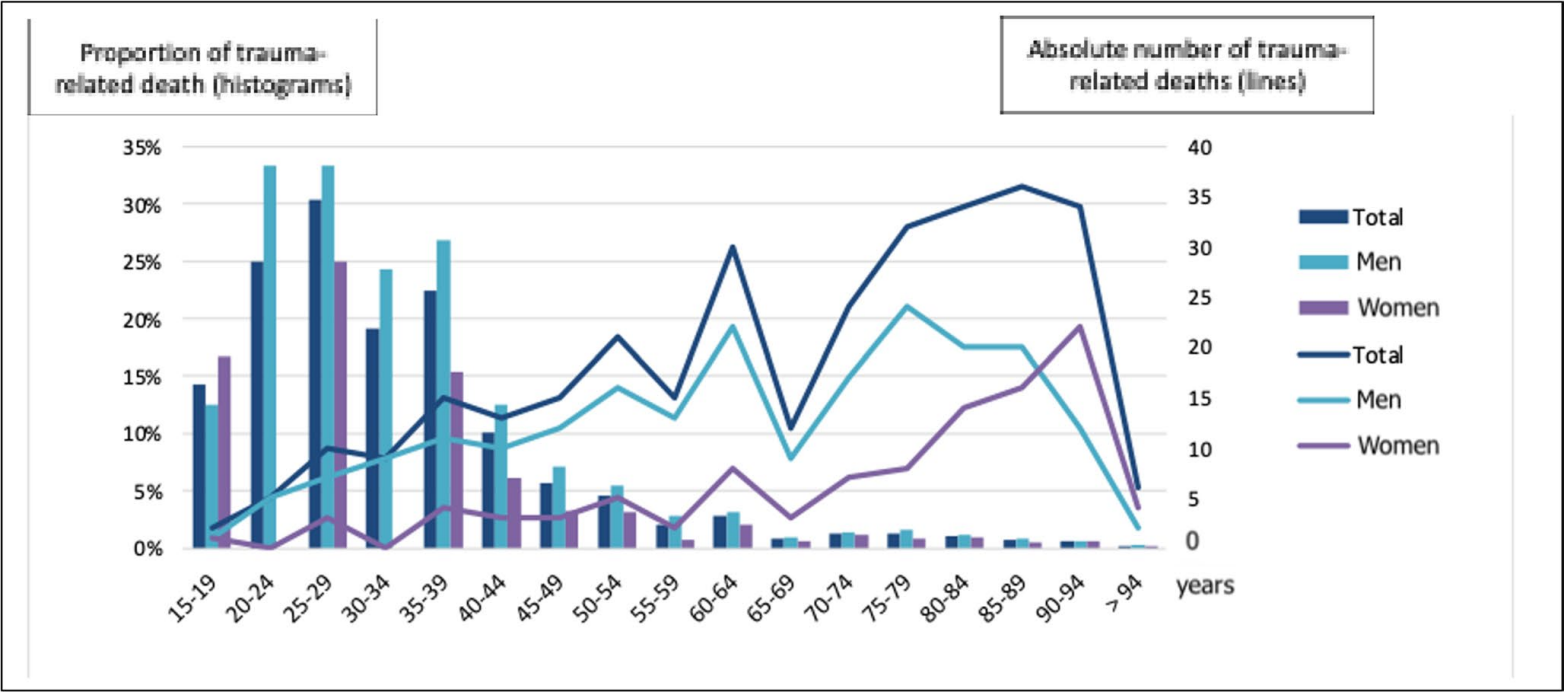
Trauma-related deaths by age group. The histogram (left axis) shows the proportion of trauma-related deaths relative to total deaths, while the line plot (right axis) represents the absolute number of trauma-related axis)

 Trauma represented an average of 1.3% of all deaths among individuals aged ≥15 years, with greater impact in younger groups: 23% of deaths in individuals aged 15–39 years, and up to 33% in men aged 20–29 years (Figure 3).

The number of years of potential life lost before age 70 (YPLL^70^) was 1,505 in 2019 and 1,655 in 2022: 236 and 417 in women, and 1,269 and 1,238 in men, respectively. Crude YPLL^70^ rates were 58 and 80 per 100,000 women, and 320 and 261 per 100,000 men, in 2019 and 2022, yielding overall rates of 188 and 166 per 100,000 inhabitants aged 15–70 years [18].

### ASA-PS and age correlation

**Fig. 4:**
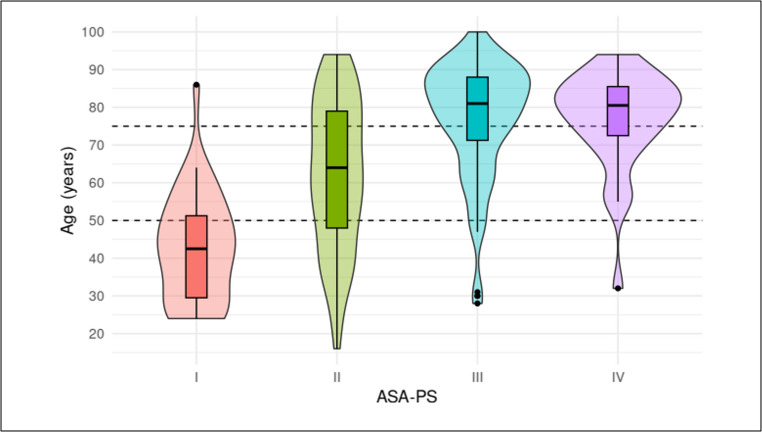
Violin plots illustrating the relationship between Age and ASA-PS score. Dotted line indicates age categories used in this study

The ASA-PS score was strongly associated with age (Fig. 4). Patients classified as ASA-PS I (12%, *n* = 36) had a median age of 43 years (P25-P75: 29–52), ASA-PS II (37%, *n* = 115): 64 years (48–79), ASA-PS III (36%, *n* = 114): 81 years (71–88); and ASA-PS IV (10%, *n* = 30): 81 years (72–86). In 5% (*n* = 18) the ASA-PS score was unavailable.

 Age categories were therefore defined according to the distribution observed in relation to ASA-PS. Categorization by ASA-PS did not yield additional insights relevant to the study objectives (Supplementary Table 2).

### Mechanism and type of injury

Blunt injuries accounted for 94% (*n* = 295) of deaths, while penetrating injuries represented 6% (*n* = 18). The most frequent mechanisms were low-energy falls (41%, *n* = 128), high-energy fall (30%, *n* = 93), pedestrian run-overs (7%, *n* = 23), car/truck collisions (5%, *n* = 15), motorcycle accidents (5%, *n* = 15), crush injuries (3%, *n* = 8), and bicycle accidents (1%, *n* = 4).

Other traffic incidents represented 1% (*n* = 3). Firearm injuries (2%, *n* = 6) and stab wounds (2%, *n* = 7) were less frequent. Overall, road traffic accidents accounted for 19% (*n* = 60) of deaths.

Significant differences by age group were observed: among individuals aged 15–50 years, falls from height (46%, *n* = 32) and traffic accidents (35%, *n* = 24) predominated; among those aged 51–75 years, low-energy falls (30%, *n* = 33) and falls from height (38%, *n* = 41) were most frequent; and among those aged > 75 years, simple falls accounted for 68% (*n* = 92) of deaths (Table 2).

**Table 2:** Summary of overall study findings and results stratified by age group. For simplicity, the categories “firearm” and “bladed weapon” were combined as “violence,” and all road traffic–related mechanisms were grouped as “traffic accidents.” AIS: Abbreviated Injury Scale; ISS: Injury Severity Score

Characteristics	Overall N-313^1^	15-50 N=69^1^	51-75 N=109^1^	>75 N=135^1^	p-value^2^
Sex					<0.001
Male	210(67%)	55(80%)	82(75%)	73(54%)	
Female	103(33%)	14(20%)	27(25%)	62(46%)	
Injury type					0.11
Blunt	295(94%)	65(94%)	99(91%)	131(97%)	
Penetrating	18(5.8%)	4(5.8%)	10(9.2%)	4(3.0%)	
Mechanism					<0.001
Traffic accident	60(19%)	24(35%)	19(17%)	17(13%)	
Violence	13(4.2%)	3(4.3%)	9(8.3%)	1(0.7%)	
Low-energy fall	128(41%)	3(4.3%)	33(30%)	92(68%)	
High-energy fall	93(30%)	32(46%)	41(38%)	20(15%)	
Other	19(6.1%)	7(10%)	7(6.4%)	5(3.7%)	
Medico-legal etiology					<0.001
Suicide	90(29%)	26(38%)	44(40%)	20(15%)	
Unintentional	216(69%)	39(57%)	64(59%)	113(84%)	
Homicide	6(1.9%)	3(4.4%)	1(0.9%)	2(1.5%)	
Unknown	1	1	1	0	
Physiopathological cause of death					<0.001
Traumatic brain injury	147(47%)	22(32%)	43(39%)	82(61%)	
Massive hemorrhage	91(29%)	29(42%)	35(32%)	27(20%)	
Multiple trauma	44(14%)	13(19%)	22(20%)	9(6.7%)	
Other	31(9.9%)	5(7.2%)	9(8.3%)	17(13%)	
Number of severely injured body regions(AIS>2)					<0.001
0	5(1.6%)	1(1.4%)	1(0.9%)	3(2.2%)	
1	165(53%)	19(28%)	55(50%)	91(67%)	
2	62(20%)	18(26%)	20(18%)	24(18%)	
3	44(14%)	17(25%)	17(16%)	10(7.4%)	
4	33(11%)	14(20%)	12(11%)	7(5.2%)	
5	4(1.3%)	0(0%)	4(3.7%)	0(0%)	
Severely injured head & neck	252(81%)	54(78%)	89(82%)	109(815)	0.9
Severely injured face	11(3.5%)	3(4.3%)	6(5.5%)	2(1.5%)	0.3
Severely injured thorax	148(47%)	49(71%)	57(52%)	42(31%)	<0.001
Severely injured abdomen	82(26%)	29(42%)	33(30%)	20(15%)	<0.001
Severely injured extremities	78(25%)	27(39%)	27(25%)	24(18%)	0.003
Severely injured vertebro-medullary	31(9.9%)	6(8.7%)	15(14%)	10(7.4%)	0.2
ISS	30(25, 66)	59(41, 75)	36(25, 75)	25(20, 33)	<0.001
NISS	48(34, 75)	66(57, 75)	57(34, 75)	35(27, 50)	<0.001

^1^n(%); Median(Q1, Q3)

^2^Pearson's Chi-squared test with simulated p-value(based on 2000 replicates); Kruskal-Wallis rank sum test

### Medico-legal intent

The medico-legal intent was predominantly unintentional (69%, *n* = 216), followed by suicide (29%, *n* = 90) and homicide (2%, *n* = 6), with significant age-related differences. Among individuals aged 15–50 years, suicides accounted for 38% (*n* = 26) and unintentional deaths for 57% (*n* = 39). In the 51–75-year group, the proportions were 40% (*n* = 44) and 59% (*n* = 64), respectively. In those older than 75 years, unintentional deaths were clearly predominant, representing 84% (*n* = 113).

### Anatomical distribution and severity

Severe injuries (AIS > 2) most frequently involved the head and neck (81%, *n* = 252) and thorax (47%, *n* = 148), followed by abdomen (26%, *n* = 82), extremities (25%, *n* = 78), and face (4%, *n* = 11). Severe spinal cord injuries occurred in 10% (*n* = 31). Differences between age groups were significant for thoracic, abdominal, and extremity injuries, which mainly affected the 15–50 year group (71%, 42%, and 39%, respectively).

**Fig. 5:**
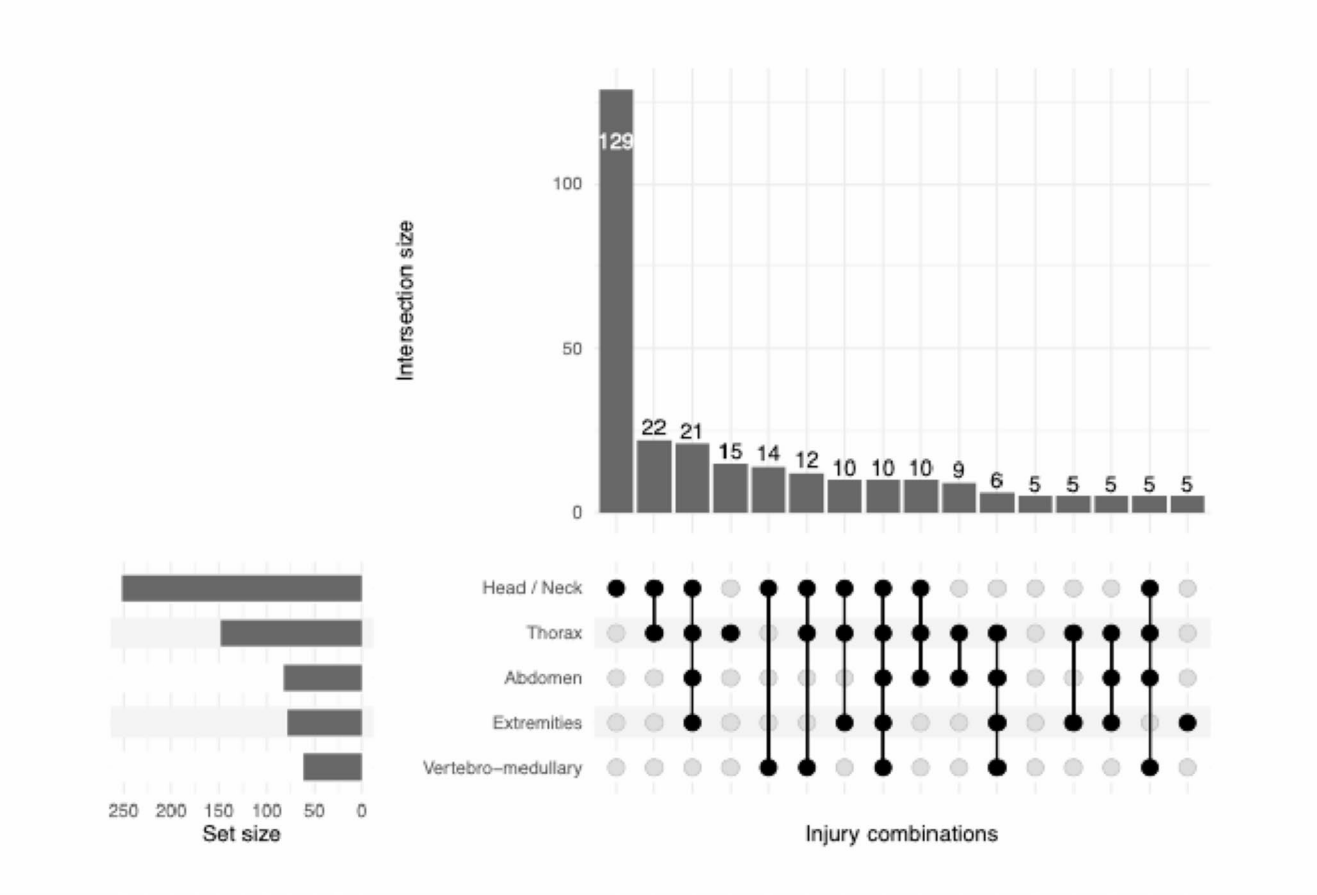
Combinations of severely injured body regions (AIS >2) among victims. Combinations with fewer than five cases were excluded

Overall, 53% (*n* = 165) had a single severely affected region; 20% (*n* = 62) had two; 14% (*n* = 44) three; 11% (*n* = 33) four; and 1% (*n* = 4) five. Only 2% (*n* = 5) had no injury with AIS > 2. Among cases with a single severely affected region, 78% (*n* = 129) involved the head and neck, and 29% (*n* = 90) combined head–neck and thoracic injuries (Fig. 5).

### Injury Severity Scores

The median ISS was 30 (P25–P75: 25–66) and the median NISS 48 (34–75), with significant variation across age groups. Among individuals aged 15–50 years, the median ISS was 59 (41–75) and the NISS 66 (57–75); in those aged 51–75 years, the median ISS was 36 (25–75) and the NISS 57 (34–75); and in patients older than 75 years, the median ISS was 25 (20–33) and the NISS 35 (27–50).

 Patients with ISS >50 accounted for 30% (n= 94) of deaths; those with ISS 20–50 for 52% (n= 164); and those with ISS < 20 for 18% (n= 55). An inverse relationship with age was observed: 55% (n = 38) of those aged 15–50 had ISS >50, compared with 11% (n = 15) of those >75. 

**Fig. 6:**
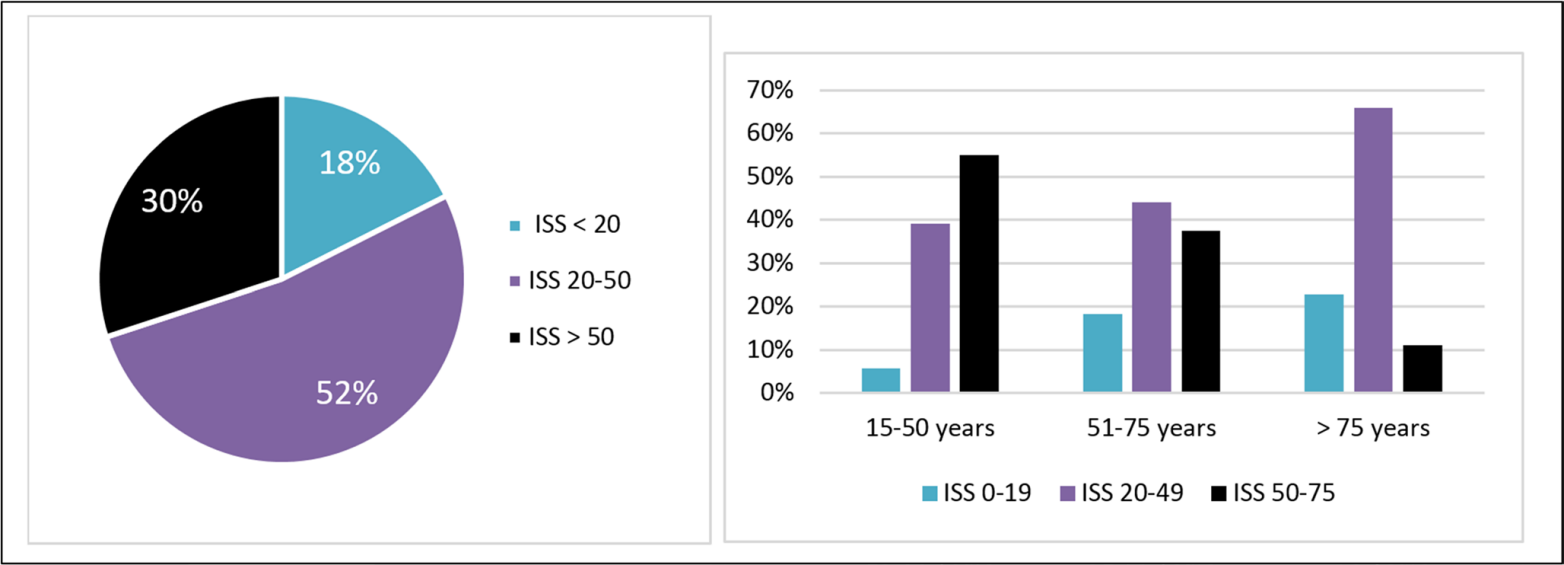
Categorized Injury Severity Score (ISS) (left) and ISS distribution by age group (right)

 Conversely, deaths with ISS <20 represented 6% (n = 4), 18% (n = 20), and 23% (n = 31) in t he respective age groups (Figure 6).

### Physiopathological causes of death

**Fig. 7:**
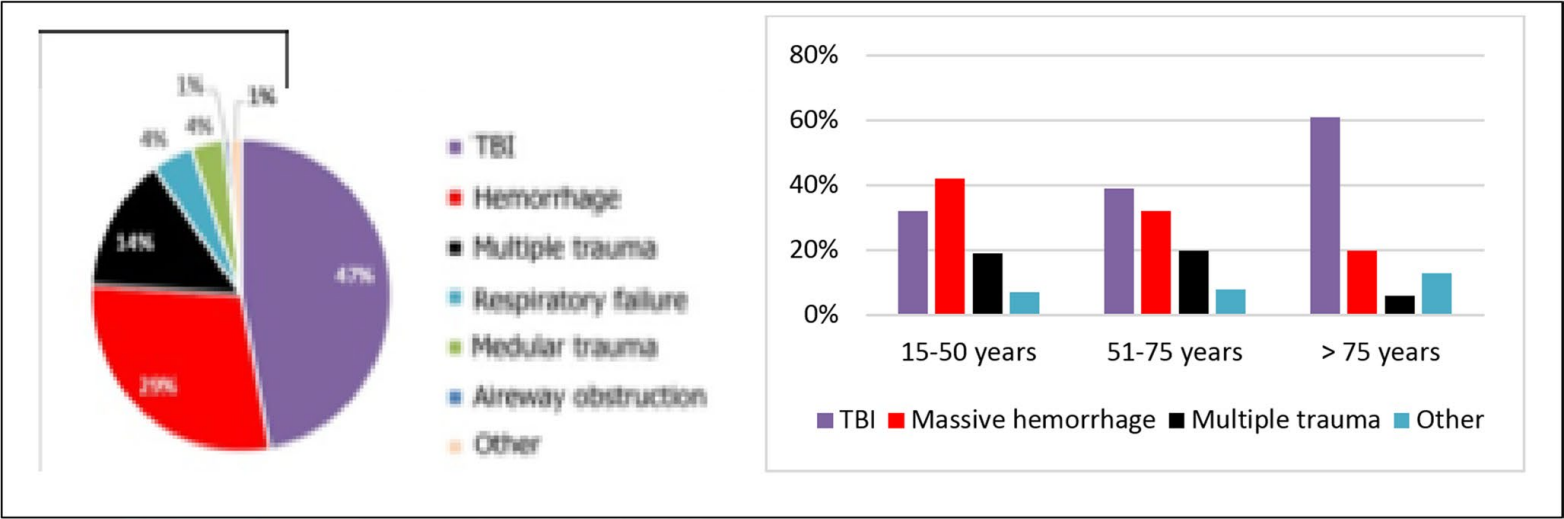
Physiopathological causes of death (left) and distribution by age group (right). TBI: traumatic brain injury

The leading physiopathological causes of death were traumatic brain injury (47%, *n* = 147), massive hemorrhage (29%, *n* = 91), and multiple trauma (14%, *n* = 44). By age group, massive hemorrhage predominated among individuals aged 15–50 years (42%, *n* = 29), followed by traumatic brain injury (32%, *n* = 22) and multiple trauma (19%, *n* = 13). In the 51–75-year group, traumatic brain injury was the main cause (39%, *n* = 43), and in those older than 75 years, it was even more frequent (61%, *n* = 82) (Fig. 7).

Detailed associations between physiopathological cause of death and number of severely injured regions are presented in Supplementary Table 3.

## Discussion

Trauma ranked as the third leading cause of years of potential life lost before age 70 (YPLL^70^) in Biscay in both 2019 and 2022, following bronchial and lung cancer and ischemic heart disease. Among men, this position remained stable, while in women it rose from seventh to fourth place, after bronchial and lung cancer, breast cancer, and brain tumors, according to mortality reports from the Basque Government Department of Health [18]. The YPLL^70^ method was used to facilitate comparison with other causes of death reported by the Basque Government. However, given the very high life expectancy in this region [20], the true burden in terms of years of life lost is likely even greater. These findings highlight the dual impact of trauma: it remains the leading cause of death among young people and a major contributor to mortality among older adults.

By encompassing the full spectrum of injury severity, this study reveals a high proportion of deaths due to low-energy trauma (falls < 3 m), particularly among elderly patients. Age groups were defined based on ASA-PS classification, a well-established independent predictor of mortality in adult trauma patients [17]. This pattern has been widely reported in Europe [11, 21] and reflects population aging, frailty, and the challenge of prehospital triage in the absence of high-energy mechanisms [22, 23].

The predominance of unintentional trauma underscores the low crime rate in the region [24], whereas the significant proportion of suicides highlights the growing impact of mental health issues [25]. Although data from the Epidemiological Surveillance Unit of the Biscay Territorial Delegation show a marked reduction in traffic accident–related mortality—from 4,610 deaths in 1991 (5.26 YPLL per 1,000 inhabitants) to 965 in 2016 (1.02 YPLL, age-standardized to the European population) [26, 27]—traffic incidents remain an important cause of death, especially among individuals under 65 years and vulnerable road users such as pedestrians, cyclists, and motorcyclists.

Analysis of the affected anatomical regions provides additional clinical insight: most victims presented a single severely injured region, challenging the traditional assumption that severe trauma necessarily implies polytrauma—a concept increasingly regarded as obsolete. The head–neck and thoracic regions were most frequently affected, either independently or in combination, confirming the high clinical complexity associated with trauma care [28, 29].

Few European studies include prehospital deaths [30, 31], although the epidemiology of trauma-related mortality is periodically described in the United States and Australia to assess the evolution of trauma systems [32–34]. Despite considerable advances in organization, prevention, and care, the two leading physiopathological causes of trauma-related death—traumatic brain injury and massive hemorrhage—have persisted over time. Our European cohort reflects the same pattern. Classifying deaths according to physiopathological mechanisms adds clinical value to mortality analysis by identifying priority areas for system improvement, complementing primary prevention efforts to develop a highly responsive, equitable trauma system aligned with real patient needs.

According to previous studies [35, 36], preventable (PD) and potentially preventable deaths (PPD) typically represent 0.7–26.1% and 3–37% of trauma deaths, respectively. Following WHO and ACSCOT criteria [4], our findings suggest a considerable margin for improvement in system performance.

It is well established that the maturation of trauma systems [8] and the treatment of severely injured patients in Level I trauma centers improve survival [9]. Although diverse organizational models coexist internationally [37], little is known about which specific components contribute most to system effectiveness [38]. Our findings suggest that distinct physiopathological profiles may require adapted care pathways. Optimizing trauma systems to accommodate these different patient trajectories—from young polytraumatized adults to frail elderly patients with low-energy injuries—could enhance both efficiency and equity in trauma care delivery.

### Limitations

This study has the inherent limitations of a retrospective observational design. Data were obtained from multiple sources, some not fully digitized. Nevertheless, the use of the autopsy database from the Forensic Pathology Service of the Basque Institute of Legal Medicine ensured exhaustive inclusion of all trauma victims, as all violent deaths undergo forensic investigation.

There is currently no universal consensus on the definition of preventable trauma deaths [31, 39, 40]. Multidisciplinary expert panels are considered the most reliable assessment method. Neither ISS nor the probability of survival (Ps) perfectly correlates with preventability, and deaths occurring after hospital discharge are particularly difficult to classify due to confounding factors such as comorbidities and natural causes [41]. In this study, forensic experts classified the underlying cause of death as violent even when the immediate cause was a later complication. In the absence of an expert review panel, ISS-based categorization was applied in accordance with WHO and ACSCOT guidelines, and results should therefore be interpreted as an approximation of the system’s potential for improvement rather than as exact estimates of preventable mortality.

Autopsy reports were crucial for determining injury severity (AIS, ISS, NISS) and physiopathological cause of death, including in out-of-hospital cases. Although discrepancies between hospital-based AIS coding and autopsy findings have been reported [42], cross-referencing multiple data sources allowed a comprehensive and accurate depiction of trauma-related mortality in Biscay.

Finally, although the sample size limits generalization to broader populations, the exhaustive nature of the registry provides a highly representative picture of the regional reality.

## Conclusion

This study provides a comprehensive characterization of trauma-related mortality in Biscay, a European region with an intermediately mature trauma care system. Two major epidemiological patterns were identified: the predominance of elderly patients and the substantial contribution of low-energy falls to trauma-related deaths.

Our findings reveal two distinct clinical profiles. Younger individuals typically die from high-energy mechanisms resulting in severe injuries, whereas an increasing number of older and frail adults die following low-energy trauma associated with less severe anatomical damage. Traumatic brain injury and massive hemorrhage remain the leading physiopathological mechanisms of death across all age groups.

While it is well established that treatment of severely injured patients in Level I trauma centers improves outcomes, the coexistence of different physiopathological profiles suggests the need to adapt trauma care pathways accordingly. Optimizing the system to address both high-energy and low-energy trauma could improve efficiency, ensure equitable access to appropriate care, and enhance patient outcomes.

Overall, these results underscore the importance of re-evaluating trauma organization from both clinical and structural perspectives. Integrating these dual patient profiles into trauma system planning and response strategies will help align trauma care with the needs of a demographically changing population and advance toward a more evidence-based, multidisciplinary, and equitable model of care.

## Supplementary Information

Below is the link to the electronic supplementary material.

Supplementary Material 1 (PNG 278 KB)

Supplementary Material 2(PNG 345 KB)

Supplementary Material 3 (DOCX 13.1 KB)

## Data Availability

The datasets used and/or analyzed during the current study are available from the corresponding author on reasonable request.

## Abbreviations

AIS: Abbreviated Injury Score
ASA-SP: American Society of Anesthesiologist Physical Status
ASCOT: American College of Surgeons Committee on Trauma
CEIm-E: Research Ethics Committee on Medicinal Products of Euskadi
CMR: Crude mortality rate
EMS: Emergency Medical Service
EsTC: Essential Trauma Care
GBD: Global Burden of Disease
HEMS: Helicopter Emergency Medical Service
IBM-SPSS: International Business Machines Corporation Statistical Package for the Social Sciences
INE: Spanish National Statistics Institute
ICCA: IntelliSpace Critical Care and Anesthesia Patient Data Management System
ICD: International Classification of Diseases
ISS: Injury Severity Score
NISS: New Injury Severity Score
NPD: Non-preventable death
PD: Preventable death
PPD: Potentially preventable death
Ps: Probability of survival
TBI: Traumatic brain-injury
YLL: Years of Life Lost
YPLL: Years of Potential Life Lost
WHO: World Health Organization
