# Supplementary figures and images for: Trauma-related mortality in a European region with an intermediately mature trauma system: a comprehensive population-based analysis
Source: Eur J Trauma Emerg Surg. 2026 Jan 13;52(1):9. doi: 10.1007/s00068-025-03043-x (PMC12799718; doi:10.1007/s00068-025-03043-x)

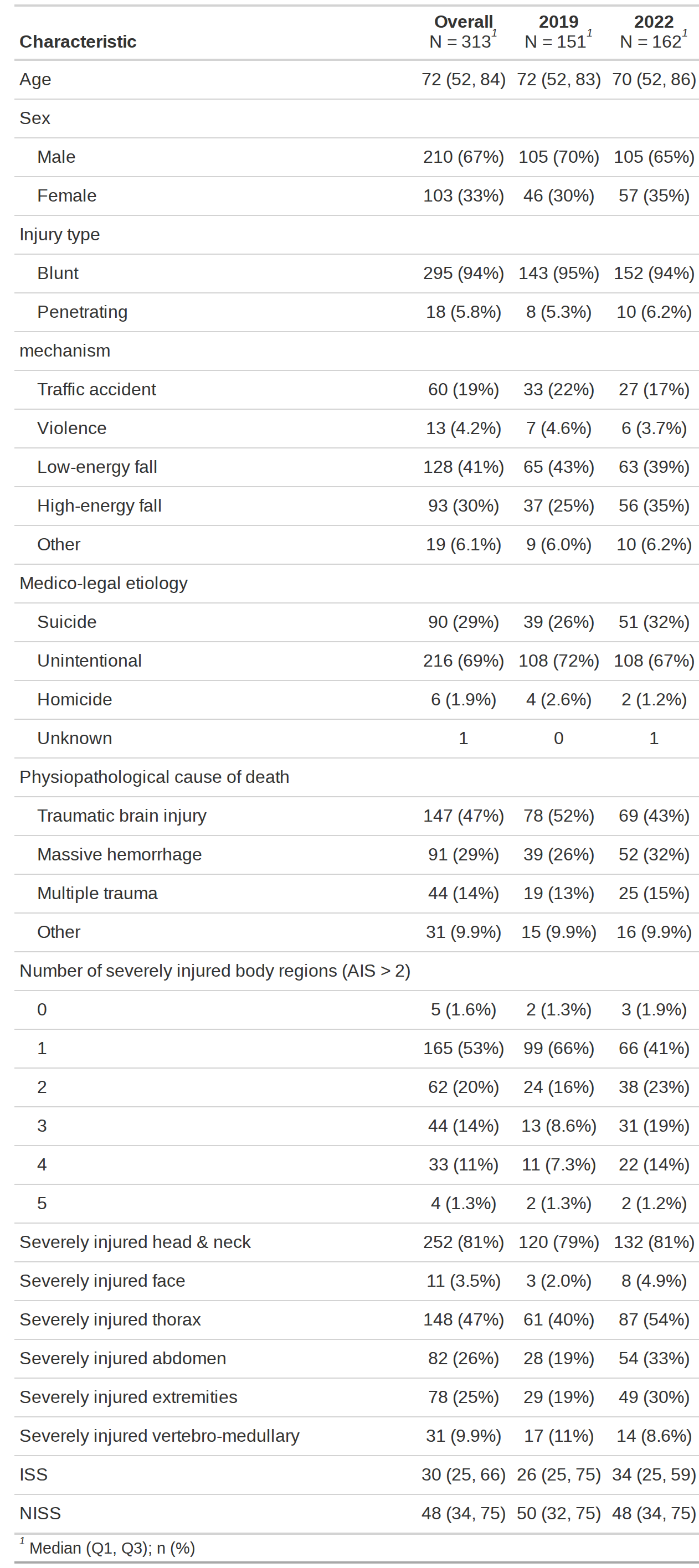

Supplement: Supplementary file 1 — Supplementary Material 1 (PNG 278 KB) [file 68_2025_3043_MOESM1_ESM.png]

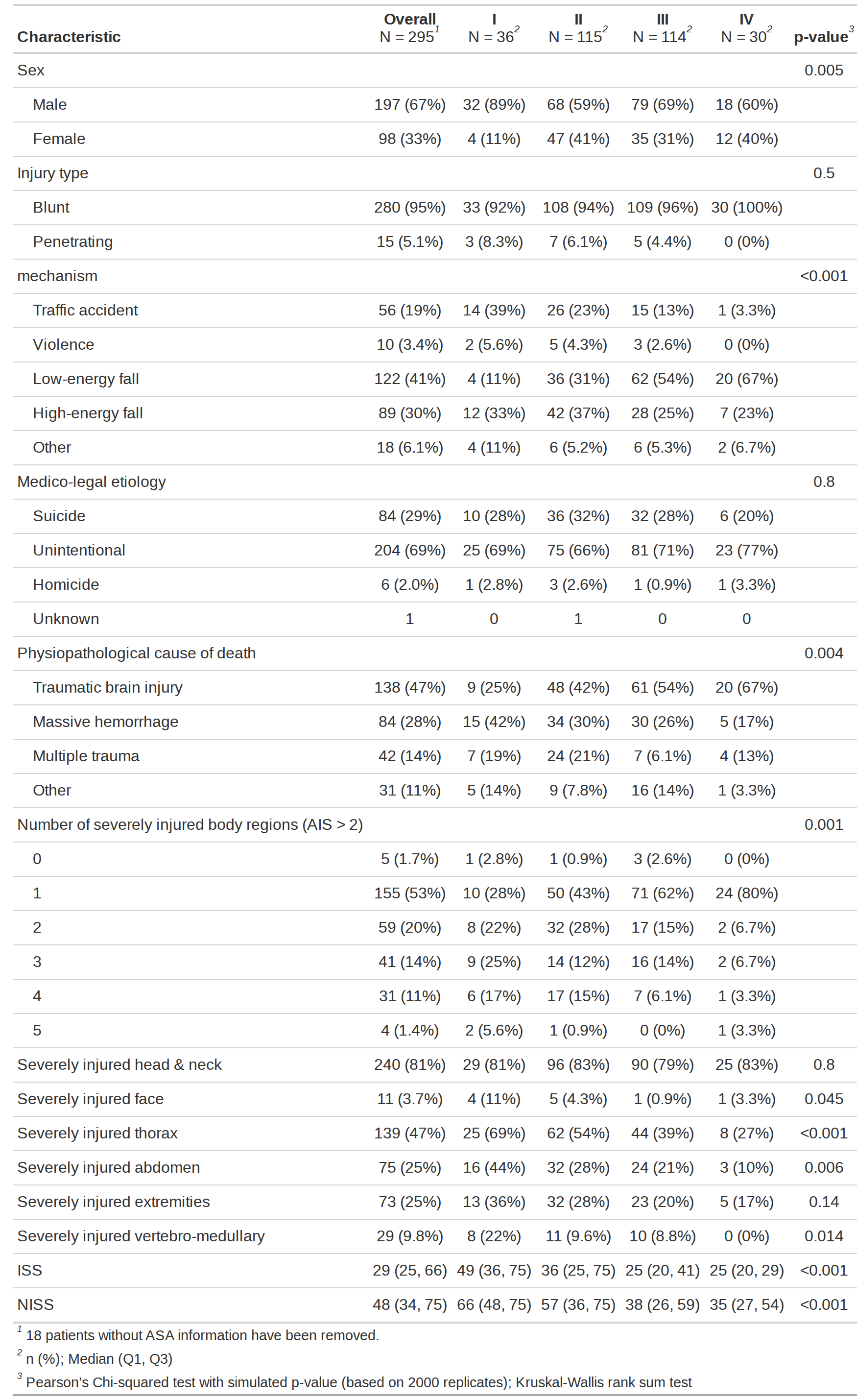

Supplement: Supplementary file 2 — Supplementary Material 2(PNG 345 KB) [file 68_2025_3043_MOESM2_ESM.png]
