# Supplementary material for: Trauma-related mortality in a European region with an intermediately mature trauma system: a comprehensive population-based analysis
Source: Eur J Trauma Emerg Surg. 2026 Jan 13;52(1):9. doi: 10.1007/s00068-025-03043-x (PMC12799718; doi:10.1007/s00068-025-03043-x)
Supplement: Supplementary file 3 — Supplementary Material 3 (DOCX 13.1 KB) [file 68_2025_3043_MOESM3_ESM.docx]

|  | | Multiple trauma | TBI | Massive hemorrhage | Other | Total |
| --- | --- | --- | --- | --- | --- | --- |
| Number of severely injured regions  (AIS >2) | 0 | 0 | 1 (20%) | 1 (20%) | 3 (60%) | 5 |
|  | 1 | 6 (4%) | 117 (71%) | 21 (13%) | 21 (13%) | 165 |
|  | 2 | 12 (19%) | 21 (34%) | 25 (40%) | 4 (6%) | 62 |
|  | 3 | 12 (27%) | 6 (14%) | 23 (52%) | 3 (7%) | 44 |
|  | 4 | 13 (39%) | 2 (6%) | 17 (52%) | 1 (3%) | 33 |
|  | 5 | 1 (25%) | 0 | 3 (75%) | 0 | 4 |

Supplementary Table 3: Association between the number of severely injured body regions and the primary cause of death. TBI: Traumatic Brain Injury. AIS: Abbreviated Injury Score.
